# Current Approaches to Epigenetic Therapy

**DOI:** 10.3390/epigenomes7040023

**Published:** 2023-09-30

**Authors:** Ekaterina D. Griazeva, Daria M. Fedoseeva, Elizaveta I. Radion, Pavel V. Ershov, Ivan O. Meshkov, Alexandra V. Semyanihina, Anna S. Makarova, Valentin V. Makarov, Vladimir S. Yudin, Anton A. Keskinov, Sergey A. Kraevoy

**Affiliations:** 1 Federal State Budgetary Institution, Centre for Strategic Planning and Management of Biomedical Health Risks of the Federal Medical Biological Agency, Pogodinskaya Str., 10, Building 1, Moscow 119121, Russia; 2 Federal State Budgetary Institution “N.N. Blokhin National Medical Research Center of Oncology” of the Ministry of Health of the Russian Federation (N.N. Blokhin NMRCO), Kashirskoe Shosse, 24, Moscow 115478, Russia; 3 Federal State Budgetary Scientific Institution, Research Centre for Medical Genetics, Moskvorechye, 1, Moscow 115522, Russia

**Keywords:** epigenetics, histones, DNA, modulation, pharmacology, clinical trials, innovational drugs, gene therapy

## Abstract

Epigenetic therapy is a promising tool for the treatment of a wide range of diseases. Several fundamental epigenetic approaches have been proposed. Firstly, the use of small molecules as epigenetic effectors, as the most developed pharmacological method, has contributed to the introduction of a number of drugs into clinical practice. Secondly, various innovative epigenetic approaches based on dCas9 and the use of small non-coding RNAs as therapeutic agents are also under extensive research. In this review, we present the current state of research in the field of epigenetic therapy, considering the prospects for its application and possible limitations.

## 1. Introduction

Epigenetic regulation of gene expression implies changes in gene activity without altering the coding sequence. This process depends on the balance of enzymes that catalyze reversible modifications of histones and DNA molecules [1]. Disruption of this balance may trigger the development of various diseases [2,3,4,5,6]. Currently, new approaches to correcting epigenetic mistakes are being actively developed due to progress in molecular biology methods. Indeed, NGS, as well as methylation assays, allowed us to identify new molecular targets for epigenetic therapy. Also, the boost in understanding of non-coding RNA biology contributed to progress in the treatment of certain diseases.

The prime reason for epigenetic mistakes is the dysregulation of histone- and DNA-modifying enzyme activity. To correct aberrant DNA and chromatin modifications, the following approaches are being proposed: First, low-molecular-weight (LMW) inhibitors of epigenetic regulators are being extensively studied. Despite the relative ease of use, the main drawback of LMW is its low specificity and increased risk of side effects. However, the development of CRISPR/Cas9 technology helps to address such a problem by increasing editing specificity. The core of this approach is dCas9 (dead Cas9), a catalytically inactive Cas9 that is unable to introduce double-strand DNA breaks but retains guide RNA binding activity. In therapeutic approaches, dCas9 can serve as a targeting platform for various effector proteins [7]. Indeed, dCas9 fusions with either transcriptional activators or repressors allow for the regulation of target gene expression. Next, the use of non-coding RNAs (ncRNAs) (siRNA, miRNA, etc.) empowers the degradation or posttranscriptional silencing of specific mRNA [8], which may be useful for the therapy of malignancies associated with dysregulation in oncogene expression [9]. All the existing epigenetic therapy approaches share problems of target delivery, off-target effects, and immunogenicity [10,11]. Given all the limitations, very few epigenetic drugs have been introduced into clinical practice so far; moreover, further research is needed in the epigenetic therapy field. In this review, we discuss the current state of research on epigenetic therapy in terms of prospects and limitations.

## 2. Pharmacotherapeutic Approach for Epigenome Modulation

To date, the use of LMW as an inhibitor of epigenetic regulators is the most developed approach in epigenetic therapy. There are several LMW-based epigenetic drugs in clinical practice, and in this chapter of the review, we consider the most striking examples of them [12].

**Figure 1:**
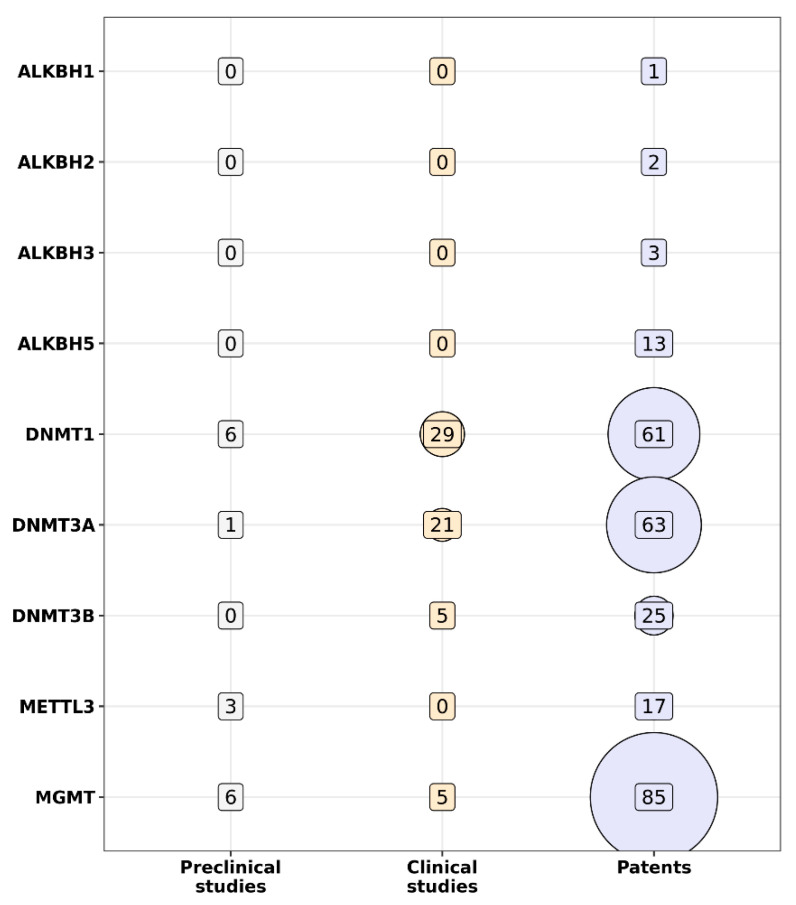
Panoramic view of drug development in relation to targeting DNA-modifying enzymes ALKBH (alkB homolog 1, histone H2A dioxygenase); DNMT (DNA methyltransferase); METTL3 (methyltransferase 3, N6-adenosine-methyltransferase complex catalytic subunit); MGMT (O^6^-methylguanine-DNA methyltransferase). The vertical numbers indicate the number of clinical and preclinical trials, as well as the number of patients involved.

**Figure 2:**
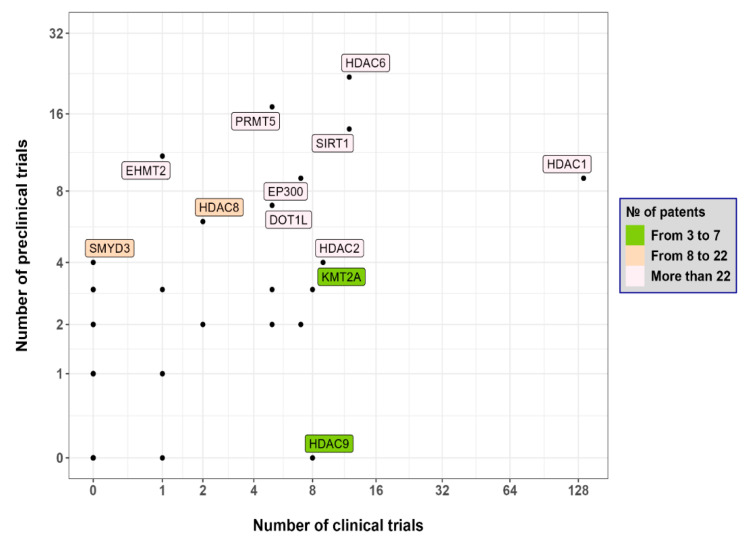
Panoramic view of drug development in relation to targeting histone-modifying enzymes The *x*-axis represents the number of clinical trials, and the *y*-axis represents the number of preclinical trials. DOT1L (DOT1-like histone lysine methyltransferase); EHMT2 (euchromatic histone-lysine N-methyltransferase 2); EP300 (histone acetyltransferase p300); HDAC (histone deacetylase); KMT2A (lysine methyltransferase 2A); PRMT5 (protein arginine methyltransferase 5); SIRT1 (deacetylase sirtuin-1).

To collect contemporary data for the last five years on preclinical and clinical trials of LMW-based epigenetic drugs, we used the Cortellis (Clarivate Analytics, Philadelphia, PA, USA) database. The database contains information on multiple patents on epigenetic drugs. Figure 1 and Figure 2 illustrate the current state of DNA- and histone-modifying enzyme-based drug development. Quite a few of the patents consider the use of LMW-based inhibitors of epigenetic regulators.

As such, LMW targets the NAD-dependent deacetylase sirtuin-1 (SIRT1), which is mentioned in 175 patents. Next, inhibitors of O6-methylguanine-DNA methyltransferase (MGMT), histone acetyltransferase p300 (EP300), DNA methyltransferase 1 (DNMT1), DNA methyltransferase-3-β (DNMT3B), histone deacetylase 1 (HDAC1), and histone deacetylase 6 (HDAC6) were cited in 85, 72, 61, 63, 60, and 66 patents, respectively. In addition, a few more clinical trials are being conducted, namely clinical trials of DNMTs and HDAC inhibitors (see Table 1). At the same time, many epigenetic inhibitors are being studied in the early phases of clinical trials alone or in combination with conventional therapy (Table 1). For example, three clinical trials are devoted to the study of the safety and efficacy of the DNMT inhibitors guadecitabine (SGI-110) [13] and azacytidine (Vidaza) [14]. Also, several clinical trials of HDAC inhibitors are being conducted, e.g., NCT01997840 (active status), NCT04231448, and NCT04674683 (recruiting status). As for LMW combination with conventional therapy, the striking example is the use of Chidamide [15], which is being studied in seven clinical trials in the III and IV phases. The next example is the use of O^6^-benzylguanine, an O^6^-alkylguanine-DNA alkyltransferase (AGT) inhibitor, in combination with standard therapy, which slows down the progression of glioblastoma and gliosarcoma in comparison with temozolomide treatment [16]. As for azacitidine, a single cohort study by Ruan and colleagues first demonstrated that the oral form of azacitidine in combination with the CHOP regimen (Cyclophosphamide, Doxorubicin, Oncovin and Prednisolone) showed good effectiveness [17]. In this case, the overall objective response rate was 85% (n = 20), and moreover, 55% of patients showed a complete response to treatment. However, not all combinations of epigenetic drugs with conventional treatment show superior efficacy. For example, in phase II/III of clinical trial NCT02472145, the use of a combination of the anti-CD123 (interleukin 3 receptor) monoclonal antibody talakotuzumab with the DNMT inhibitor decitabine was not more efficient than the treatment with decitabine alone in patients with acute myeloid leukemia (AML) [18]. The next example, guadecitabine, is a prodrug that is more active in vivo than decitabine. In AML treatment, 8 of 56 patients responded to guadecitabine, which demonstrated an increase in overall survival from 7.1 to 17.9 months compared to decitabine [19,20]. Another therapeutic scheme for AML that is currently in phase I of a clinical trial (EudraCT No. 2018-000482-36) uses iadademstat (ORY-1001), a selective inhibitor of lysine-specific histone demethylase (KDM1A) [21]. This protocol demonstrated good safety as well as clinical activity. To assess the effectiveness of iadademstat in combination with etoposide and cisplatin in patients with recurrent small cell lung cancer, a study called CLEPSIDRA (EudraCT No. 2018-000469-35) was initiated. The study showed that iadademstat alone reduces tumor growth by 90%, moreover, in combination with chemotherapy, it increases progression-free survival by up to 50% [22].

Another example is seclidemstat, a KDM1A inhibitor, alone or in combination with chemotherapy, which is going to be studied in a phase I clinical trial (NCT03600649) with the enrollment of 50 patients with relapsed or refractory sarcomas. Preclinical studies of seclidemstat showed significant inhibition of tumor growth in neoplasms with KDM1A overexpression [23].

Tumor-associated histone deacetylases (HDAC1–HDAC10 isoforms) are considered promising molecular targets for cancer therapy [24]. Table 2 shows the list of approved and experimental drugs aimed at both isoforms (e.g., abexinostat) as well as broad-spectrum HDAC inhibitors (e.g., vorinostat and pracinostat). It is interesting to note that a number of statin-related drugs are viewed as HDAC inhibitors; however, the molecular mechanisms underlying their effects are only just beginning to be explored. For example, Lin and colleagues showed that statins block the activity of HDAC2 indirectly through the induction of histone H3 acetylation in the promoter region of the p21 gene [25]. Bridgeman and colleagues suggested that statins do not directly affect the activity of HDACs and HATs (histone acetyltransferases) since the degree of histones H3 and H4 acetylation did not change when compared to the control. At the same time, there was some increase in the activity of DNA methyltransferases [26].

LMW HDAC inhibitors can also affect the activity of other proteins. Based on the chemical structure of nine different HDAC inhibitors (vorinostat, pracinostat, atorvastatin, mocetinostat, valproic acid, bufexamac, trichostatin A, abexinostat, and fingolimod) as well as using the DRUDIT web-based tool [27], 29 non-specific drug targets were predicted, 15 of which took part in metabolic and signaling pathways (Table 3). Therefore, the use of HDAC inhibitors may result in systemic non-specific (off-target) effects on various cellular pathways [28]. The problem of low LMW selectivity and off-target effects may be solved by the development of alternative therapeutic approaches. As such, new protocols that use effector proteins, ncRNAs, as well as biotherapeutic agents (small proteins, DARPins (designed ankyrin repeat proteins), and monoclonal antibodies) should be developed. Unfortunately, only a limited number of such innovative drugs are gradually being introduced into clinical practice.

### Oncometabolites and Metabolic Rewiring

The expression of metabolic enzymes was demonstrated to be altered in several cancer types [30]. A high frequency of somatic mutations in *IDH1* (isocitrate dehydrogenase 1), *FH* (fumarate hydratase), as well as *SDH A*–*D* and *F* (succinate dehydrogenase) genes is observed in gliomas, hepatobiliary cancers, neuroendocrine carcinomas, renal cell carcinomas, and melanomas [31]. These mutations lead to enzymatic activity deregulation and the accumulation of so-called "oncometabolites." Oncometabolites can activate oncogenic signaling cascades [32], induce deregulation of epigenetic patterns, resistance to alkylating agents, collagen maturation impairment, inhibition of protein succination, etc. [33,34,35].

Since oncometabolites cause global metabolic rewiring in cancer cells [34], pharmacological targeting of enzymes with altered activity should be considered in cancer therapy [36,37]. Recent achievements in the development of inhibitors of mutant IDH1 and IDH2 are discussed in the reviews by W. Tian and co-authors [38] as well as Issa & DiNardo [39]. The ClinicalTrials.gov database contains information on studies of metabolic reprogramming therapy in cancer patients. Table 4 contains summary information on the most prominent examples of metabolite reprogramming in cancer therapy. Thus, the pharmacotherapeutic "management" of cellular metabolite levels demonstrates high potential in cancer therapy.

## 3. Innovative Molecular and Genetic Approaches to the Modulation of Epigenetic Regulation

### 3.1. CRISPR/Cas9

CRISPR/Cas9 is a genome editing platform based on the Cas9 endonuclease, which introduces double-strand breaks in DNA sequences complementary to the corresponding guide RNA (gRNA). Despite the accuracy of targeting, gRNAs may have sites of incomplete homology. In this case, the nuclease introduces DNA breaks in random places, which can lead to undesirable consequences, so-called "off-target effects."

The problem of non-specific DNA breaks may be solved by using the modified Cas9 protein, dCas9. The dCas9, carrying substitutions D10A and H841A, is unable to introduce double-strand DNA breaks but retains the gRNA binding activity [7]. dCas9 can be fused in-frame to various effector proteins, providing a platform for their targeting to the locus of interest. Different epigenetic modulators—either transcriptional activators or repressors—may act as such effectors. In early works, dCas9 was fused with either p300 (human acetyltransferase catalytic core p300), p65 (endogenous transcription factor p65 (NFkB subunit), p65 with HSF1 (heat shock factor 1), or VP64 (herpesvirus transcription factor) [40,41,42,43]. These activation systems, so-called CRISPRa (CRISPR activation), such as dCas9-p300, may act by histone acetylation in target sites, or alternatively, like dCas9-p65, may directly activate genes by recruiting transcription factors. For example, the activating effect of VP16 [44] is based on the recruitment of the RNA polymerase preinitiation complex to the transcription start site, followed by the activation of transcription of the target gene. However, the activation capability of VP16 alone is very low, so to cope with this limitation, most CRISPRa protocols use VP16 multimers (VP48, VP64, VP160, and VP192) in combination with other activators [45,46]. One such example is dCas9-VPR, which is composed of VP64-p65-RTA, where VP64 consists of four VP16 subunits and RTA is an Epstein-Barr virus transcription factor. The dCas9-VPR complex is significantly more effective than early variants [47,48].

Despite the visible activation effect, the early dCas9-based activation systems share the same limitation and require multiple gRNAs targeted to extended genome regions to achieve reliable activation. The more recent CRISPR-Cas9 technologies such as SunTag, Scaffold, Casilio, SAM, and TREE allow target genes to be activated with fewer or even a single gRNA. For example, dCas9-SunTag uses dCas9 fused to the GCN4 peptide array, which, in turn, binds scFv-GCN4-fused effector proteins [49,50]. Thus, SunTag allows the concurrent use of numerous effector domains to enhance epigenetic activation. Alternately, complexes of dCas9 with an RNA scaffold work in a different way: the modified guide RNA contains aptamer sequences (MS2, PP7) that are recognized by corresponding RNA-binding proteins (MCP, PCP). Transcriptional activators fused to these proteins are recruited to the dCas9-scaffold-RNA complex and enhance target gene transcription activation [51]. As an improvement of this, the dCas9-Casilio uses shorter Casilio aptamers in the gRNA sequence, enhancing gRNA stability and efficiency [52]. The next approach, SAM, is a combination of dCas9 fused to transcription activators with scaffolding to enhance target gene activation [53,54]. Finally, the TREE system combines the SunTag and scaffold, allowing for up to 32 copies of VP64 or p65-HSF1 to be recruited [55].

CRISPRi (CRISPR interference), the use of dCas9 to repress target gene expression, employs the fusion of functional domains of repressor proteins with dCas9. For instance, the KRAB (Krüppel-associated box) domain of several repressor proteins, such as EZH2 (Enhancer of Zeste 2 Polycomb Repressive Complex 2 subunit), is widely used in CRISPRi approaches [56,57,58]. dCas9-KRAB can recruit histone deacetylases and methyltransferases to the promoters and enhancers of target genes [59]. Being recruited, the histone methyltransferases introduce corresponding histone methyl marks, leading to heterochromatin formation and transcription repression [60,61]. Just like CRISPRa, CRISPRi suffers from some limitations. For example, the repression effect of individual use of either dCas9-KRAB or dCas9-EZH2 is temporary. To cope with this problem and achieve constant transcription repression, the combination of dCas9-KRAB/EZH2 with either dCas9-DNMT3A-3L or Cas9-SunTag-DNMT3A may be used [62,63,64,65].

Another repressive system is dCas9-KRAB-MeCP2, in which MECP2 (methyl-CpG binding protein 2) recruits histone demethylases and deacetylases independently of KRAB [66,67]. The MECP2 protein binds to 5-methylcytosines in CpG islands of promoters of target genes, repressing their transcription. The dCas9-KRAB-MeCP2 repression efficiency is significantly higher than for dCas9-KRAB [67]. Another system, dCas9-LSD1, may also be utilized for transcriptional repression. LSD1 (KDM1A, lysine-specific demethylase-1) removes methyl groups from H3K4me1/2, an active chromatin mark [68], which in turn leads to H3K27 deacetylation and heterochromatin formation [69].

dCas9 platforms for epigenetic regulation have some drawbacks that complicate their use in gene therapy. Firstly, the existing delivery systems have significant limitations in terms of transfer capacity. For instance, the size of the dCas9 ORF is 4.1 kb, which practically corresponds to the capacity of AAV vectors (4.7 kb). Alternatively, the packaging capacity of lentiviral vectors is about 8 kb, but they are mainly used for ex-vivo therapy. The second limitation of dCas9-based therapy is the duration of its therapeutic effect. The span of the dCas9-based epigenetic regulation effect is currently unknown; moreover, it may vary in each particular case. Next, off-target effects also hinder the translation of dCas9-based technologies into clinical practice. The degree of off-target effects should be studied in each case; moreover, the harm of off-target effects may outweigh the benefit of the therapeutic protocol. The development of dCas9-based gene therapy methods is a new direction in science, and no wonder it has many unresolved questions that take time to answer. So far, only a very limited number of dCas9-based technologies have reached the stage of preclinical and clinical trials. For example, only one clinical trial of the CRISPRa drug CRD-TMH-001 for the treatment of DMD (Duchenne Muscular Dystrophy) has been FDA-approved (NCT05514249). The drug is based on rAAV9 delivery of the dCas-VP65 transgene for upregulation of cortical dystrophin. Unfortunately, in this trial, the potential benefit of the therapy outweighed the risk to the patient’s immune system. Nevertheless, many studies have already been carried out on cell cultures and animal models. In the following part of this review, we will provide examples of dCas9-based epigenetic regulation in research on various therapy protocols.

The use of dCas9 in cancer therapy is an area of great interest. One such example is the epigenetic activation of the *PTEN* (phosphatase and tensin homolog) gene [48]. Abnormal *PTEN* expression is observed in many cancer types; moreover, even minor changes in *PTEN* expression affect the prognosis of many highly aggressive malignancies [70]. The decrease in *PTEN* expression may be a result of a variety of factors, including mutations and epigenetic silencing. In the latter case, the CRISPRa protocols may be used. In the work of Moses et al., *PTEN* expression activation was achieved by dCas-VPR in TNBC and SK-MEL-28 cells. The authors showed that *PTEN* activation significantly suppresses AKT, mTOR, and MAPK signaling and reduces cell migration and colony formation in the presence of B-Raf and PI3K/mTOR inhibitors [48]. Thus, dCas9-mediated *PTEN* activation may provide an alternative approach to treating aggressive cancers resistant to current therapeutic protocols.

One more noteworthy example of gene expression epigenetic regulation in cancer therapy is the study on hepatocellular carcinoma cells by Wang et al. Hepatocellular carcinoma is the most common primary liver cancer. In the work of Wang et al., the GRN (granulin) was chosen as a therapeutic target. Increased *GRN* expression is observed in many neoplasias, especially in hepatocellular carcinoma. The authors used dCas9-KRAB to epigenetically target *GRN* in hepatic carcinoma cells and demonstrated its effect on Hep3B carcinoma cells [71]. The next example of dCas9-based epigenetic therapy is the study of FSHD (facioscapulohumeral muscular dystrophy) treatment. The disease is caused by abnormal epigenetic modifications in the D4Z4 DNA tandem repeat array, which is located in the subtelomeric region of chromosome 4q35 [72,73]. Each repeat contains *DUX4* (Double Homeobox 4) ORF. During early development, the DUX4 protein upregulates the expression of many genes whose aberrant expression in adult skeletal muscle can lead to pathology. In the work of Himeda et al., the authors showed that targeting either the promoter or the first exon of *DUX4* by dSaCas9 fused with epigenetic repressors significantly reduced the expression level of *DUX4* in myocytes from biopsies of FSHD patients [72]. Another interesting example is the activation of *SCN1A* (Sodium Voltage-Gated Channel Alpha Subunit 1) gene expression by dCas9-fused epigenetic activators. The *SCN1A* encodes for the α-subunit of the voltage-gated sodium channel Nav1.1. Mutations in the *SCN1A* are associated with Dravet Syndrome (DS, severe myoclonic epilepsy in infancy), a drug-resistant epileptic encephalopathy. The main genetic cause of DS is haploinsufficiency of the *SCN1A* gene. Indeed, SCN1A^+\−^ mice develop neurological symptoms, including severe epilepsy, behavioral changes, and premature death [74]. Given that one copy of the *SCN1A* is still functional, stimulation of its expression may lead to an increase in Nav1.1 production and, as a result, symptom relief. In recent work, Colasante and colleagues demonstrated stable activation of *SCN1A* transcription in P19 mouse teratocarcinoma cells using dCas9-VP160. The authors also showed the ability of dCas9-VP160 to modulate *SCN1A* activity in primary neurons by increasing the level of Nav1.1. Moreover, the authors revealed that dCas9-VP64 efficiently stimulates *SCN1A* expression in GABAergic interneurons in vivo when delivered by means of AAV9 [75].

Recently, dCas9-based approaches have begun to be studied in the context of genomic imprinting disorders. A striking example is the use of dCas9 for epigenetic regulation in Prader-Willi syndrome (PWS). PWS, a typical genomic imprinting disorder, is a complex neurobehavioral disease with a birth incidence of 1/12,000 to 1/32,000 [76,77]. The disease is caused by a deficiency in gene expression on the 15q11–q13 locus of the paternal chromosome [76]. Gene expression in this region is regulated by an imprinting center (PWS-IC), which is located upstream of the paternally expressed *SNRPN* (small nuclear ribonucleoprotein polypeptide N) gene. PWS-IC is methylated on the maternal chromosome, repressing the PWS candidate genes, but is not methylated on the paternal chromosome. 23 genes are mapped in the 15q11–q13 region, including the *SNORD116* cluster and 15 other genes. 12 of them are subjected to genomic imprinting and are only expressed on the paternal chromosome. In PWS patients, the expression of these genes is absent due to molecular defects in the 15q11–q13 region of the paternal chromosome. The modulation of the epigenetic state of the imprinting domain can restore the PWS genes’ expression on the maternal chromosome, providing a therapeutic effect in PWS patients. For example, it is possible to use dCas9-LSD1 to demethylate H3K9me2 in the PWS-IC of the maternal chromosome, resulting in the reactivation of *SNRPN* or *SNORD116* expression [78]. Besides PWS, the treatment of Silver-Russell syndrome (SRS) is an interesting example of a possible use of dCas9-based approaches. SRS is a clinically and genetically heterogeneous condition characterized by severe intrauterine and postnatal growth restriction caused by the decreased expression of the *IGF2* (insulin-like growth factor 2) gene [79,80]. DNA hypomethylation of the imprinting control center between the *H19* and *IGF2* genes (*H19* differentially methylated region; *H19*-DMR) on the paternal chromosome can be found in 35–50% of SRS patients. *IGF2* and *H19* are reciprocally imprinted genes and are regulated by the methylation of *H19*-DMR [81,82]. *IGF2* is expressed only on the paternal allele. Meanwhile, *H19* is expressed only on the maternal allele. *H19*-DMR contains four highly conserved CG-rich CTCF binding sites that can block methylation spreading [83]. In the paternal allele, CpG methylation within CTCF binding sites abolishes CTCF binding and results in a loss of enhancer-blocking activity, thereby allowing *IGF2* expression. Conversely, hypomethylation of the paternal *H19*-DMR allows the binding of CTCF, leading to the expression of both *H19* alleles and the downregulation of *IGF2*. All this eventually results in growth retardation. The correction of methylation patterns is a promising strategy for patients with SRS. In the study conducted by Horii et al., the authors developed an SRS model using dCas9-SunTag fused to (GFP)-TET1CD (ten-eleven translocation hydroxylase). The authors demethylated the *H19*-DMR locus in mouse embryonic stem cells and in fertilized mouse eggs [84]. This work is a good demonstration of the possibility of dCas9 usage for target modification of the methylation pattern.

Apart from the therapy of genomic imprinting disorders, dCas9-based approaches can be used for potential therapy of repeat expansion diseases, such as fragile X syndrome (Martin-Bell Syndrome, Fragile X syndrome, FXS). FXS is an X-linked cognitive disorder with a range of neurological and psychiatric problems. The main cause of FXS is the loss of *FMR1* (Fragile X Messenger Ribonucleoprotein 1) expression during neurodevelopment [85]. The *FMR1* silencing is caused by hypermethylation of its promoter due to the CGG trinucleotide repeat expansion to the *FMR1* 5′-UTR. In healthy individuals, *FMR1* contains approximately 6–44 repeats, while in FXS patients, more than 200 repeats can be found. FMRP (Fragile X mental retardation protein), encoded by *FMR1*, is an RNA-binding protein expressed in neurons that controls protein synthesis in developing synapses and plays a key role in synaptic plasticity maintenance [86]. Lui et al. showed that binding of dCas9-TET to CGG repeats caused a significant decrease in *FMR1* promoter methylation as well as a partial restoration of FMRP expression in human cells [87]. Demethylation of the CGG repeat region increased both H3K27 acetylation and H3K4 trimethylation and, moreover, decreased H3K9 trimethylation in the *FMR1* promoter region, leading to *FMR1* expression reactivation. This study suggested that targeted demethylation of CGG repeats reactivated *FMR1* in a variety of FXS models using iPSCs as well as in vitro-derived FXS neurons. Demethylation of CGG repeats resulted in the conversion of the heterochromatin into the active state of the upstream *FMR1* promoter. Therefore, the results provide the first direct evidence that the de-methylation of CGG repeats is sufficient for *FMR1* reactivation. It is important to know that methylation editing reversed the abnormal electrophysiological phenotype of FXS neurons and that FMRP expression in the edited neurons remained adequate in vivo [87,88].

### 3.2. Non-Coding RNAs

The use of ncRNAs is another convenient tool for epigenetic regulation. In recent years, several classes of ncRNAs have been discovered: miRNA, siRNAs, long noncoding RNAs (lncRNA), piwi-interacting RNAs (piRNAs), circular RNAs (circRNAs), etc. [8]. Despite the fact that the first attempts to therapeutically use ncRNAs started immediately after the discovery of RNA interference (RNAi) in 1998 [89], the first RNAi-based drug, patisiran (ONPATTRO), was approved only in 2018 [90,91]. Table 5 contains a summary of siRNA-based drugs that have already been FDA-approved or are currently in clinical trials.

The development of ncRNA-based drugs is complicated by the following issues: low stability of RNA molecules, target delivery difficulties [93], possible toxicity, and immunogenicity [8,94]. The first two major problems with ncRNA-based therapy are the low stability of RNA molecules and their rapid clearance from the system. When injected into the blood, unmodified RNAs are rapidly degraded by serum RNases and also excreted from the body by secretion in the renal tubules. This leads to a short half-life of RNA drugs when systemically administered. To cope with the stability problem, an RNA molecule may be chemically modified on either the ribose or the phosphodiesterase bond [95,96]. Ribose modifications include 2′-O-(2-Methoxyethyl), 2′-O-Methyl, 2′-LNA (Locked Nucleic Acid), 2′-F (2′-fluoro). The phosphodiesterase bond modifications are usually associated with the replacement of phosphate groups by thiophosphate ones [97]. These days, all FDA-approved RNA aptamers (pegaptanib), inhibitory antisense oligonucleotydes (mipomersen, nusinersen, inotersen), and siRNA (patisiran) contain various ribose modifications [98]. However, some RNA modifications may activate the cellular part of the immune system or impede ncRNA function. This applies particularly to the methylation of nitrogen bases, especially cytosine. Moreover, for siRNA, the complete or partial nucleotide substitution for 2′-O-Methyl results in the loss or reduction of inhibition activity [95]. One explanation for such a functional impediment would be the blocking of siRNA-RISC interaction by the 2′-O-methyl group [99]. The introduction of 2′-F into all siRNA nucleotide positions has a similar effect. At the same time, the combination of phosphodiesterase bond thiophosphate modification and the 2′-F significantly increases the toxicity of RNA-based drugs [100]. Modification with LNA (locked nucleic acid) increases the stability of the duplex and suppresses the immune response; however, it was shown that LNA introduction into the first 5′-RNA nucleotide completely inhibits RNA interference [101].

Another challenge for the therapeutic use of RNA-based drugs is target delivery. Due to their small size and negative charge, RNA molecules are unable to cross the cell membrane by themselves. To facilitate penetration through the cell membrane, cationic polymers enriched with positively charged amino groups should be utilized. Polyethyleneimine (PEI) is an example of a polymer that is widely used for siRNA delivery. PEI can not only bind siRNA but also act as a proton sponge capable of destroying the endosome membrane and facilitating the release of the siRNA complex into the cytoplasm. However, in preclinical and clinical trials, the siRNA-PEI complexes induce inflammation, liver necrosis, thrombus formation, and affect lung endothelium [102]. Polylysine-based nanoparticles and branched polymeric substances (dendrimers) can also be used for siRNA delivery. Dendrimers have a regular three-dimensional structure and usually carry amino acid residues, facilitating entry into the cell. Next, polysaccharides constitute another class of siRNA delivery facilitator molecules; among them are chitosan, dextran, hyaluronan, and hyaluronic acid [103]. The important advantage of such molecules is their low toxicity and immunogenicity, as well as their enhanced biodegradability. Conjugates of N-acetylgalactosamine (GalNAc) with siRNAs are actively utilized for RNA-based drug delivery into liver cells through interaction with the asialoglycoprotein receptor [104]. In addition, lipid nanoparticles (LNPs) are often used to deliver siRNAs [105].

Immune response is the next issue in ncRNA-based therapeutic approaches. In the cytoplasm, double-stranded RNAs (dsRNAs) are recognized by cellular defense systems as foreign RNAs, causing activation of TLRs (Toll-like receptors), NLRs (NOD-like receptors), and RLRs (RIG-I-like receptors), as well as stimulating an interferon response [106,107,108]. For example, TLR7 and TLR8 recognize the 5′-UGU-3′ motif inside the RNA sequence [109,110], activating the interferon response. Next, the 5′-GUCCUUCAA-3′ motif causes TLR7-dependent increased cytokine production [111]. In general, immune response activation via TLR7 and TLR8 is caused by the presence of closely spaced ribose and uracil molecules. Thus, immune response activation is highly dependent on the sequence and chemical structure of siRNA molecules [112,113]. Endogenous miRNAs may also act as TLR agonists, as has been shown for most miRNAs secreted by tumor cells. MiR-21 and miR-29a are able to cause macrophage activation through interaction with TLR-7 and TLR-8 receptors, leading to NF-kB activation and proinflammatory cytokines production (e.g., TNF and ILs) [94]. The let7 miRNA is also able to activate TLR-7 receptors in macrophages, microglial cells, and neurons, which also indicates the presence of GU-rich regions in the RNA sequence [114]. One of the possible approaches to reducing siRNA toxicity and immunogenicity is long-term drug administration in minimal doses. For example, the delivery of anti-*KRAS* oncogene siRNA mimetics into cells of inoperable pancreatic cancer resulted in the release of the RNA-based drug within 12 weeks (NCT01676259) [9].

siRNA and miRNA are the main players in post-transcriptional gene silencing. They act by targeting complementary mRNA sequences, mainly in the 3′UTR. The key difference between the actions of siRNA and miRNA is their target molecule degradation mechanism. While siRNAs act through direct degradation of the target RNA transcript, miRNAs mainly provide translational silencing. However, miRNA can facilitate the indirect degradation of transcripts via target mRNA deadenylation and decapping, followed by subsequent RNA lysis with exonucleases [115].

The following options exist for siRNA drug design: delivery of either siRNA precursors or mature siRNA [90]. siRNA precursors are usually 26–28 bp long and have a hairpin-shaped region corresponding to the 5′-end of the leading (antisense) strand [116]. The mature siRNAs form dsRNA complexes of 21 and 23 nt long with a 2-nt unpaired region at the 3′ end. For activation, siRNA binds to Argonaute proteins, then the sense siRNA strand is removed, followed by the antisense strand binding to the active protein complex [117]. For proper binding, siRNAs must be long enough to interact with RISC complex machinery, and siRNA silencing efficiency is significantly decreased for siRNAs shorter than 19 bp in length [118].

As for miRNA-based drug development, the following issues should be considered: First, miRNAs have a complementary seed region of 6–7 bp long. Such a small complementarity area causes miRNA molecules to act on several targets simultaneously, resulting in an uncontrolled impact on gene expression [119]. To overcome this problem, a cocktail of several miRNAs at very low concentrations that are targeted to the same mRNA can be used. As an example, a combination of miR-34a and miR-15a/16 was applied to non-small cell lung cancer cells, causing cell cycle arrest via *CCNE1* and *CCND3* gene knockdown [120]. Currently, there are no approved miRNA-based drugs. Moreover, none of the miRNA-based drugs have reached phase III clinical trials. An important problem in miR-NA-based drug implementation is off-target effects [121].

Another class of ncRNA molecules, circRNAs (circular RNAs), has recently been intensively studied [122,123]. circRNAs appear to play an important role in gene expression regulation. Changes in circRNA expression have been shown to be associated with various tumors, neurodegenerative diseases, and metabolic diseases [124]. circRNAs perform different functions in the cell. They can act as molecular sponges that adsorb both miRNAs and transcription factors, regulating their functional activity. For example, ciRS-7, the first discovered circRNA, contains more than 70 conserved miR-7 binding sites [123]. Both endogenous and artificial circRNAs may be used as miRNA sponges for changing pathogenic miRNA activity levels in the regulation of certain diseases [124]. Several circRNA sponges, complementary to oncogenic miR-21 and miR-122, have been developed [125,126,127], but currently, there are no circRNA-based drugs in preclinical or clinical trials. The use of circRNAs as therapeutic agents is a promising area of drug development. Due to their structure, circRNAs are more resistant to RNase degradation than linear RNAs [128], which allows the use of lower doses of circRNA-based drugs. Also, circRNAs demonstrate lower immunogenicity even without any modifications [129]. Thus, circRNAs are an attractive area for drug development.

Apart from being miRNA sponges, circRNAs are able to adsorb some transcription factors. Protein adsorption is achieved by introducing protein-binding sites into the circRNA sequence [130]. Furthermore, circRNAs in the cell often act as RNA aptamers that facilitate protein complex assembly. The creation of artificial circRNA aptamers may also be useful for therapeutic protein delivery as well as for controlling their activity [122]. Next, circRNAs can be used as templates for peptide synthesis. This is achieved by introducing an internal ribosome entry site into the RNA sequence along with an ORF containing start and stop codons. Protein synthesis, in this case, occurs via the rolling circle mechanism [131]. In this case, much higher levels of protein expression can be achieved than when translated from linear transcripts. However, without termination signals, such translation can trigger multimeric repetitive peptide motif synthesis and also cause undesirable toxic effects [128].

Apart from small RNA molecules, synthetic analogues of miRNA molecules are currently being developed. Synthetic or modified miRNA molecules can be used as miRNA inhibitors. Table 6 summarizes current data on clinical trials of such drugs.

## 4. Conclusions

Epigenetic aberrations and pathogenesis processes are tightly interlinked. These days, epigenetic aberrations may be subject to pharmacological correction. According to the scale of current clinical trials, one may assume that the introduction into clinical practice of an extended list of epigenetic drugs is coming soon. Particularly, epigenetic drugs targeting cellular enzymes that reversibly modify chromatin are being intensively studied. Several epigenetic therapy approaches are being developed. Among them, the dCas9-based approach is a promising direction that is still in its infancy. More research is needed for a better understanding of the opportunities and limitations of epigenetic therapy.

## Figures and Tables

**Table 1:** A spectrum of clinical trials of drugs targeting proteins involved in epigenetic regulation.

Protein Name	Drug	ID Trial (www.ClinicalTrials.gov, Accessed on 16 September 2023)	Phase	Disease
DNA methyltransferase DNMT	Guadecitabine, SGI-110	NCT03206047	I/II	Platinum-Resistant Fallopian Tube Carcinoma, Platinum-Resistant Ovarian Carcinoma, Platinum-Resistant Primary Peritoneal Carcinoma, etc.
		NCT01261312	I/II	Myelodysplastic Syndromes (MDS), Acute myeloid leukemia (AML), Chronic Myelomonocytic Leukemia (CMML)
		NCT02197676	II	Myelodysplastic Syndromes (MDS),
	Decitabine	NCT02472145	II/III	Acute myeloid leukemia (AML)
		NCT04051996	II	Acute myeloid leukemia (AML)
	Azacitidine, CC-486	NCT03542266	II	Peripheral T-cell lymphoma (PTCL)
Histone acetyltransferase EP300	Inobrodib, CCS1477	NCT03568656	I/II	Metastatic Castration-Resistant Prostate Cancer, Metastatic Breast Cancer, Non-small Cell Lung Cancer, Advanced Solid Tumors
Histone acetyltransferase DOT1L	Pinometostat	NCT03701295, NCT03724084	I/II	Acute myeloid leukemia (AML)
Histone methyltransferase PRMT5	Pemramethostat, GSK3326595	NCT04676516	II	Early stages of breast cancer
Histone demethylase KDM1A	Tranylcypramine	NCT02717884	I/II	Acute myeloid leukemia (AML) Myelodysplastic Syndrome
	Seclidemstat	NCT03600649	I/II	Ewing Sarcoma, Myxoid Liposarcoma, Sarcoma, Soft Tissue, Desmoplastic Small Round Cell Tumor, etc.
	Iadademstat	NCT05546580	I	Acute myeloid leukemia (AML)
		NCT05420636	II	Small cell lung cancer (SCLC), Neuroendocrine Carcinoma
NAD-dependent deacetylase SIRT1	Selisistat, SEN0014196	NCT01521585	II	Huntington’s disease
	Chidamide, HBI-8000,Tucidinostat,	ChiCTR1800017698 *	IV	Diffuse large B-cell lymphoma (DLBCL)
		ChiCTR2000034301 *	N/A	Advanced breast cancer
		ChiCTR-OIC-17011303 *	IV	Peripheral T-cell lymphoma (PTCL)
		NCT03023358	III	Peripheral T-cell lymphoma (PTCL)
		NCT04674683	III	Metastatic inoperable melanoma
		NCT04231448	III	Diffuse large B-cell lymphoma (DLBCL)
		NCT04040491	IV	Peripheral T-cell lymphoma
	Fimepinostat, CUDC-907	NCT03002623	II	Thyroid Neoplasms, Poorly Differentiated and Undifferentiated Thyroid Cancer, Differentiated Thyroid Cancer
Histone deacetylase HDAC	Givinostat	NCT01901432	I/II	Polycythemia Vera
	Ricolinostat, ACY-1215	NCT01997840	I/II	Multiple myeloma
		NCT02856568	I	Non-Resectable Cholangiocarcinoma, Recurrent Cholangiocarcinoma, Stage III Extrahepatic Bile Duct Cancer, Stage III Intrahepatic Cholangiocarcinoma, etc.
	Quizinostat	NCT01486277	II	T cell lymphoma
	MGCD-0103	NCT00358982	II	Hodgkin’s lymphoma
	Resminostat, 4SC-201	NCT00943449	II	Advanced hepatocellular carcinoma
	Entinostat	NCT00866333	II	Hodgkin’s lymphoma

* ID Trial (https://www.chictr.org.cn, Accessed on 16 September 2023).

**Table 2:** Histone deacetylase inhibitors and their status.

Protein Name	Drug Name	DrugBank ID	Status
HDAC1-3, HDAC6, HDAC8	Vorinostat	DB02546	FDA approved
HDAC1-3, HDAC6	Pracinostat	DB05223	Investigational
HDAC1, HDAC2, HDAC4, HDAC6	Atorvastatin	DB06176	FDA approved
HDAC1–3	Mocetinostat	DB11830	Investigational
HDAC2 HDAC9	Valproic acid	DB00313	FDA approved
HDAC10 HDAC6	Bufexamac	DB13346	FDA approved
HDAC7 HDAC8	Trichostatin A	DB04297	Experimental
HDAC1	Abexinostat	DB12565	Investigational
	Fingolimod	DB08868	FDA approved
HDAC2	Atorvastatin	DB06176	FDA approved
	Fluvastatin	DB01095	FDA approved
	Pravastatin	DB00175	FDA approved
	Lovastatin	DB00227	FDA approved
	Simvastatin	DB00641	FDA approved
HDAC4	CID 24836810 *	DB08613	Experimental
	CID 24836811 *	DB07879	Experimental
HDAC8	CID 3994 *	DB02565	Experimental
	CID 5287979 *	DB07586	Experimental
	CID 10379137 *	DB07350	Experimental
	CID 449096 *	DB02917	Experimental
	Cumarin 120	DB08168	Experimental

* Entry No. in the PubChem database (https://pubchem.ncbi.nlm.nih.gov/, accessed on 16 September 2023).

**Table 3:** Prediction of off-target proteins for HDAC inhibitors and their involvement in cellular processes.

KEGG Terms *	*p*-Value	Protein *
hsa00350: Tyrosine metabolism	0.0002	Amine oxidase copper containing 3 (AOC3)
		Monoamine oxidase A (MAOA)
		Tyrosinase (TYR)
		4-hydroxyphenylpyruvate dioxygenase (HPD)
hsa00360: Phenylalanine metabolism	0.0016	Amine oxidase copper containing three proteins (AOC3)
		Monoamine oxidase A (MAOA)
		4-hydroxyphenylpyruvate dioxygenase (HPD)
hsa04068: FoxO signaling pathway	0.0012	Mitogen-activated protein kinase 10 (MAPK10)
		Mitogen-activated protein kinase 9 (MAPK9)
		Serine/threonine kinase 4 (STK4)
		Serum/glucocoticoid regulated kinase 1 (SGK1)
		Sirtuin 1 (SIRT1)
hsa04024: cAMP signaling pathway	0.0052	Mitogen-activated protein kinase 10 (MAPK10)
		Mitogen-activated protein kinase 9 (MAPK9)
		Phosphodiesterase 4D (PDE4D)
		5-hydroxytryptamine receptor 1A (HTR1A)
		Phosphodiesterase 4A (PDE4A)
hsa04010: MAPK signaling pathway	0.0120	Mitogen-activated protein kinase 10 (MAPK10)
		Mitogen-activated protein kinase 9 (MAPK9)
		Calcium voltage-gated channel auxiliary subunit alpha2 delta 1 (CACNA2D1)
		Protein tyrosine phosphatase non-receptor type 7 (PTPN7)
		Serine/threonine kinase 4 (STK4)
hsa04012: ErbB signaling pathway	0.0392	Mitogen-activated protein kinase 10 (MAPK10)
		Mitogen-activated protein kinase 9 (MAPK9)
		ABL proto-oncogene 1, non-receptor tyrosine kinase (ABL1)
hsa04014: Ras signaling pathway	0.0472	Mitogen-activated protein kinase 10 (MAPK10)
		Mitogen-activated protein kinase 9 (MAPK9)
		ABL proto-oncogene 1, non-receptor tyrosine kinase (ABL1)
		Serine/threonine kinase 4 (STK4)

* Functional enrichment with KEGG (Kyoto Encyclopedia of Genes and Genomes) terms was performed using DAVID v. 6.8 [29].

**Table 4:** A list of relevant clinical trials on metabolite reprogramming in different types of cancer.

ID *	Phase	Cancer Type	Oncometabolite	Drug Combination
NCT03449901	II	Soft tissue sarcoma	Arginine	ADI-PEG20, gemcitabine, docetaxel
NCT04776889	IV	Prostate cancer (metastatic)	Cholesterol	Rosuvastatin
NCT04862260	Early Phase I	Pancreatic cancer	Cholesterol	FOLFIRINOX, ezetimibe, atorvastatin, evolocumab
NCT04164901	III	Glioma	2-hydroxyglutarate	Vorasidenib
NCT03173248	III	Acute myeloid leukemia	2-hydroxyglutarate	Ivosidenib, azacitidine

* (www.ClinicalTrials.gov**,** accessed on 16 September 2023).

**Table 5:** A spectrum of clinical trials of siRNA-based drugs.

Commercial Name	Substance	Clinical Trial No.	Target	Progress
ONPATTRO	patisiran	NCT01617967 NCT02510261 NCT04201418 NCT03997383 NCT05040373	Transthyretin (TTR)	FDA approved, long-term studies, pregnancy safety studies
LEQVIO	Inclisiran [92]	NCT05362903, NCT04929249 NCT05682378 NCT03159416 NCT05399992	Proprotein convertase subtilisin/kexin type 9 (PCSK9)	FDA approved, long-term study, combination therapy effectiveness study, extension trials
OXLUMO	Lumasiran	NCT04152200 NCT03905694 NCT03350451 NCT04982393	Hydroxyacid oxidase (OA1)	FDA approved, observational study, extension trials
GIVLAARI	givosiran	NCT04883905 NCT02452372	aminolevulinic acid synthase 1 (ALAS1)	FDA approved, combination therapy
-	Cemdisiran	NCT02352493 NCT05070858 NCT04601844 NCT05744921 NCT05133531	Complement 5	Phase I/II completed, Combination therapy trials
Anti-EpHa2 siRNA	Anti-EpHa2 siRNA	NCT01591356	ephrin type-A receptor 2 (EpHa2)	Phase I estimated in 2024
CAS3/SS3	Anti-CpG-STAT3 siRNA	NCT04995536	TLR9 receptor and signal transducer and activator of transcription 3 (STAT3)	Phase I estimated in 2024
NBF-006	Anti-KRAS siRNA	NCT03819387	*KRAS* proto-oncogene (*KRAS*)	Phase I estimated in 2024
ALN-KHK	antiKHK siRNA	NCT05761301	ketohexokinase (KHK)	Phase I/II estimated in 2025
OLX10212	Asymmetric siRNA	NCT05643118	Pathways upstream of *VEGF* (vascular endothelial growth factor)	Phase I estimated in 2024
ADX-038	Anti-PK siRNA	NCT05876312	*Prekallikrein* (PK),	Phase I estimated in 2025
SRN-001	Anti-AREG siRNA	NCT05984992	Amphiregulin (AREG)	Phase I estimated in 2024
AOC 1020	Anti-DUX4 siRNA	NCT05747924	Double homeobox 4(DUX4)	Phase I estimated in 2025
AGX148/PH-762	Anti PD-1 siRNA	NCT05902520	siRNA Modulation of PD-1	Phase I estimated in 2026

**Table 6:** A spectrum of clinical trials of miRNA mimetics and inhibitors.

Commercial Name	Substance	Clinical Trial No.	Progress
INT-1B3	miR-193a-3p mimic	NCT04675996	Phase I estimated in 2024
MRX34	miR-34a mimic	NCT01829971 NCT03033329	Terminated with adverse effects in 2017 Phase I completed in 2017
MRG-201	Remlarsen [132]	NCT03601052 NCT02603224	Phase 2 completed in 2020
SPC3649	Miravirsen [133,134]	NCT00979927 NCT00688012 NCT01200420	Phase 2 discontinued in 2021
MRG-106	Cobomarsen	NCT03837457 NCT02580552 NCT03713320	Phase 2 terminated in 2020
